# Changing the narrative: Resilience of women in STEM in sub-Saharan Africa and institutional innovations to advance equity

**DOI:** 10.1371/journal.pone.0338973

**Published:** 2026-01-21

**Authors:** Monica Fisher, Violet Nyabaro, Ruth Mendum, Sujata Ganguly, Moses Osiru

**Affiliations:** 1 Sustainable Agrifood Systems Program, International Maize and Wheat Improvement Center, Nairobi, Kenya; 2 Social Science and Impact Assessment Unit, International Centre of Insect Physiology and Ecology, Nairobi, Kenya; 3 Agricultural Sciences Global, College of Agricultural Sciences, The Pennsylvania State University, Pennsylvania, United States of America; 4 Sustainable Agrifood Systems Program, International Maize and Wheat Improvement Center, Nairobi, Kenya; 5 Regional Coordination Unit of the Regional Scholarship and Innovation Fund, International Centre of Insect Physiology and Ecology, Nairobi, Kenya; Caleb University, NIGERIA

## Abstract

Gender disparities in science, technology, engineering, and mathematics (STEM) remain pronounced in many African countries, particularly at the postgraduate level. This study explores the experiences of African women in STEM postgraduate education by integrating data from an online survey of 163 female PhD alumni from 40 African universities in 17 countries and seven focus group discussions (FGDs) with 39 current postgraduate students across three countries. Through a mixed-methods approach, we examine both the challenges women face and the factors that enable their persistence and success. Over 60% of respondents reported financial stress during their PhD, and more than half felt unprepared at the time of program entry. Yet 95% expressed confidence in their ability to succeed, reflecting strong self-efficacy despite structural barriers. In the FGDs, women highlighted the burden of caregiving responsibilities, lack of role models, and cultural norms that pressure them to prioritize family over academic careers. Contrary to common assumptions, most FGD participants preferred male supervisors, citing competitiveness or lack of support from some senior women. Despite these obstacles, participants demonstrated high levels of resilience, often driven by a passion for science and strong family support. Our findings highlight the need for family-friendly policies, structured and tailored mentoring, and flexible, gender-responsive institutional reforms to ensure more inclusive and equitable STEM postgraduate environments in Africa.

## Introduction

Women remain underrepresented in science, technology, engineering, and math (STEM) in most world regions [1]. In sub-Saharan Africa (SSA), women make up 30% of researchers in science fields, roughly the global average of 28% [1]. Data from nine flagship African universities for the 2010/11 academic year show female student enrolment in undergraduate and postgraduate STEM programs ranges from 25% (Edward Mondlane University) to 45% (Cape Town University and University of Mauritius) [2]. Women’s representation in STEM fields drops progressively moving up the education (and career) ladder, with women comprising 53% of bachelor’s degree holders, 43% of master’s graduates and just 28% of PhDs [1].

Influential commentaries and reviews stress the urgency of closing Africa’s gender gap in STEM [3,4], which is considered a major impediment to identifying effective, science-based solutions to Africa’s complex development problems. African women have long contributed to science and development, often behind the scenes and despite systemic barriers [5]. Removing existing barriers to women’s entry and advancement in STEM fields will expand the pool of scientists, by drawing on women’s untapped potential, and enhance the diversity of scientific teams. This, in turn, will lead to scientific findings that are comprehensive and robust and STEM innovations that better address the demands and circumstances of a diversity of stakeholders [3,4,6].

It is encouraging that African governments are increasingly taking actions to support women scientists in research and development. For example, the African Union (AU) declared 2015 the Year of Women’s Empowerment and adopted the Science, Technology, and Innovation Strategy for Africa 2024 [4]. To support governmental and other efforts seeking to advance women in STEM, there is a need to generate evidence on the main challenges women scientists face to their advancement, the strategies they use to overcome barriers, and institutional best practices that foster gender equity in STEM.

Research on women in STEM overwhelmingly focuses on North America and Europe, where well-documented challenges include isolation and lack of mentors in male-dominated programs, gender stereotyping, and unconscious biases, which manifest as gender-based financial and other barriers [6–15]. Research for the African context is very scarce, but on the rise, with recent review papers [5] and empirical studies, including multi-country [16–18] and individual country studies [19].

Building on the growing African literature on women in STEM, this study seeks to make four main contributions. First, like [18], we focus on the experiences of postgraduate students, a group often overlooked in favor of women working in STEM [16,17]. Second, to our knowledge, this is the only mixed-methods study for SSA that not only examines the challenges faced by women in STEM but also highlights their achievements. The quantitative data come from an online survey conducted in 2020 with 227 individuals, 72% of whom were female, who pursued a STEM PhD at a university in SSA within the last 20 years. Complementary qualitative data were collected from seven focus group discussions (FGDs) held in 2019 and 2020 with women MSc and PhD students in STEM fields at universities in Senegal, Rwanda, and Kenya.

Our third contribution is the study’s geographic breadth, with data representing 17 countries and 40 African universities. This includes smaller nations, like Senegal and Rwanda, which are making important strides in gender equity in higher education [20] but have received limited attention in a literature that has emphasized larger countries, such as Ghana, Kenya, and South Africa [16,19,21]. Even within single countries [22], significant regional differences in gendered STEM outcomes have been documented, which clarifies the need to expand research to underrepresented contexts and previously unexamined regions.

Finally, the study’s discussion section complements the empirical findings by sharing best practices for advancing gender equity in STEM that are already underway on the continent. By looking to African countries, institutions, and scholars driving progress in higher education, this study challenges the common practice of drawing lessons primarily from the Global North to address socio-economic conditions in the Global South. Instead, it positions Africa as a source of innovation and resilience.

## Materials and methods

### Study overview

This mixed-methods study draws on original data from an online survey and FGDs conducted in 2019/2020 to explore women’s experiences in STEM postgraduate programs in sub-Saharan Africa. It addresses two research questions: (1) What are the main challenges faced by women postgraduate students in STEM fields in Africa? (2) What factors enable these students to overcome the challenges they confront?

Data were collected by the study authors and trained interviewers to support research purposes and to inform recommendations for the Regional Scholarship and Innovation Fund (RSIF, https://www.rsif-paset.org) program’s gender strategy. The study applied a broad definition of STEM that includes, along with formal and natural sciences, the social sciences, specifically economics and psychology, both of which are critical to understanding applied issues such as food security and climate change.

### Research ethics

The RSIF gender study was conducted in accordance with the ethical principles outlined in the Declaration of Helsinki and approved by the Institutional Review Board of the International Centre of Insect Physiology and Ecology (icipe). Approval was granted because (a) the study was a socioeconomic investigation involving an online SurveyMonkey survey and FGDs with adult respondents (no minors, biological sampling, or animal subjects were involved); (b) the research questions and analytic methods were deemed sound; (c) the study protocol outlined strong data anonymization safeguards to protect participant privacy; and (d) informed consent was obtained from all participants prior to participation.

For the online survey, participants received a standard consent form outlining the study purpose, voluntary nature of participation, confidentiality assurances, risks and benefits, survey length, and contact information for the lead researcher. Consent was indicated electronically before respondents could proceed.

For the FGDs, verbal consent was obtained and documented by the facilitator signing on behalf of each participant, in line with ethical guidelines prioritizing participant anonymity. FGD participants also provided consent for publication of anonymized quotes. Transcripts contain no potentially identifying information. Informed consent forms for both methods are provided in the supporting information (S1 Appendix and S2 Appendix).

### Quantitative data collection and analysis

The quantitative component addressed the study’s two research questions by analyzing survey data from respondents who had pursued a STEM PhD in SSA over the previous two decades. The structured questionnaire included Likert-scale and binary response items across key themes: motivation, stressors, self-efficacy, family and societal pressure/support, student-advisor relationship, mentorship and role models, peer and institutional support, chilly climate (sexism/discrimination), sexual harassment, and suggested strategies.

The online survey, implemented in both English and French via SurveyMonkey, also collected demographic, socioeconomic, funding, and academic performance information. The instrument (see S1 Appendix) was co-developed by the lead authors and refined through pre-testing with recent African PhD graduates affiliated with icipe. As noted in the Research Ethics section, informed consent was obtained electronically before participation.

Recruitment occurred from February 21 to May 9, 2020. Given the absence of a sampling frame of STEM PhD graduates from SSA universities, participants were recruited via RSIF’s network, online postings, and partner organizations. An initial pool of 262 respondents was reduced to 227 by excluding individuals affiliated with non-SSA universities. Among the final sample of 227 individuals (163 women and 64 men), 74% had completed their PhDs in the past 10 years, and 25% in the past five years. For a map of the countries and institutions included in the survey, see Fig 3 in [18], which draws on the same dataset.

### Qualitative data collection and analysis

#### Qualitative data collection.

The study’s qualitative data were collected through FGDs conducted in December 2019 and February 2020. These included seven women-only groups, with four smaller groups (2–4 participants) and three larger groups (8–11 participants). A total of 39 female postgraduate students participated. Recruitment began on November 26, 2019, and the study concluded on February 20, 2020. Participants were enrolled in Physics or Information Technology programs at four RSIF universities in three countries across East and West Africa. While the quantitative survey focused on PhD students, the qualitative component included both MSc (75%) and PhD (25%) students to increase group sizes and capture a broader range of perspectives on the postgraduate student experience. To protect confidentiality, the participating institutions are not named.

Participants were recruited through RSIF’s established relationships with faculty at the surveyed universities. Across the three countries where FGDs were conducted, discussions were moderated by a female lecturer and two female postgraduate students from the Anthropology Department of a university in East Africa. We also engaged two experienced local qualitative researchers to assist with facilitation and notetaking. Before each discussion, participants were given detailed information about the study and the research team, offered the opportunity to ask questions, and rapport was established. Verbal informed consent was obtained prior to participation, as outlined in the Research Ethics section. The discussions were audio-recorded and supplemented by field notes from the notetakers.

Because the FGDs explored sensitive issues such as gendered power relations and discrimination, care was taken to ensure that local cultural norms shaped the research process. All facilitators were women familiar with local languages, communication styles, and social expectations. Their contextual understanding helped foster trust and encouraged participants to speak openly. The qualitative data collection lead held regular debriefs with the country teams to reflect on positionality, participant comfort, and any ethical concerns. No adverse incidents were reported.

#### Qualitative data analysis.

Qualitative data analysis began with transcription of audio recordings and handwritten notes, completed by two female postgraduate students. The study’s three lead authors conducted the subsequent analysis. A sociologist on the team provided training, coaching, and supervision in qualitative methods to the two economists.

A deductive approach was used, with coding categories developed based on the study’s two research questions and key themes from the online survey. Categories included motivation for pursuing postgraduate studies, psycho-social well-being and stressors, self-efficacy, family and societal pressure/support, role models and mentors, departmental culture, and sex-based discrimination.

In an initial virtual meeting, the team created a coding guide that defined inclusion/exclusion criteria, outlined attributes to extract from each transcript, and assigned color codes to each category. The lead and second author then independently reviewed and highlighted relevant text within each transcript. Both authors completed coding tables summarizing key patterns and overall impressions, accompanied by illustrative quotes. A second virtual meeting was held to compare results, discuss emerging themes, and agree on selected quotations for presentation.

### Weaving together the quantitative and qualitative findings

The final phase of the study involved an interpretive synthesis of the quantitative and qualitative findings. This mixed-methods approach offered advantages in terms of complementarity, as descriptive statistics from the online survey revealed overall patterns, while the FGDs added nuance by capturing the lived experiences behind these patterns.

This weaving process enhanced the relevance of our findings for both academic and policy audiences. The diversity of the author team—spanning four countries (India, Kenya, Uganda, and the United States) and four academic disciplines—further enriched the interpretation by bringing a range of perspectives to bear on the data.

## Results

### Motivation for PhD studies

**Fig 1:**
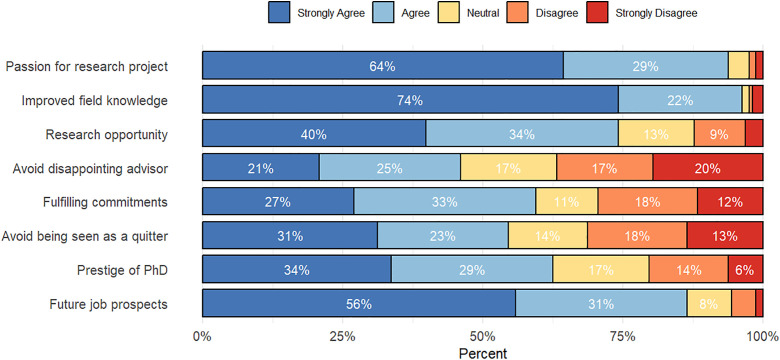
Factors motivating PhD persistence among sampled women in STEM doctoral programs in sub-Saharan Africa (*n* = 163).

The online survey included several questions to elucidate factors that motivate students to persist in their PhD programs in SSA. Fig 1 presents the percentages of responses across a 5-point Likert scale for these questions, highlighting key drivers of PhD persistence. (It should be noted that to improve readability, data labels are omitted for values of 5% or less in this and subsequent figures.) The sampled PhD alumni were most strongly motivated by the desire to improve their field knowledge, passion for their doctoral research project, and future job prospects. Although reported as less influential, factors related to social pressure, such as not wanting to disappoint their PhD advisor, and commitments, such as those to funding organizations or a current employer, also played a role.

In the FGDs, discussants spoke about their motivation to pursue rather than persist in their postgraduate studies. The findings on persistence (online survey) and initial motivation (FGDs) are complementary. When asked why they chose to pursue a career in STEM, participants in four of the seven FGDs expressed how passionate they are about their chosen field of study. Two quotes are illustrative:

“*For me it was out of passion because I have always loved the networks and IT and that is all that pushed me to come and register here and do the training.”*

*“When I landed in physics, I was so passionate. By the way everything is about physics. I tell people you cannot even have biological discoveries when you don’t have physics because those equipment without this physics concept. So, I am so passionate about it because it is very practical.”*

Many FGD participants described how an influential person in their life – a teacher, parent, or public figure –inspired them to pursue postgraduate studies in STEM.

*“Personally, I was influenced by Wangari Maathai the way she was bold.”*

*“For me it was my parents because both mum and dad were doing some science courses.”*

*“For my case when I was in secondary school, I had a good teacher in physics that made me like physics and I wanted to do electronics and reach to the university, most men teach at the center but there was one for micro biotechnology she was a woman and she was good, so I wanted to be like her.”*

It is noteworthy that some respondents mentioned public figures like Wangari Maathai while others cited family and former teachers. Building a cohort of successful scientists may require both symbolic and personal sources of inspiration.

A few respondents expressed a drive to prove oneself as a main reason for their choice to pursue postgraduate degrees in STEM, as illustrated here:

*“To me I think it is more of why can’t women do that so it is like you feel that we want to prove to other women that you can also do it and encourage women to take the technical path we have taken, at least we have mentors who mentor us.”*

This expressed desire to prove that women can be successful in a technical field implies that, at least for this respondent, women’s participation in STEM fields is still a relatively rare and new phenomenon that enjoys little support among the public.

A desire to play a role in reducing gender gaps in STEM and in the workplace generally was also expressed by some of the discussants, as in this comment:

*“For me I realized that women in technology were few in our country so I decided let me not be in a bigger category where women are doing marketing, they are doing journalism so I decided to be in information technology to bridge the gap.”*

This remark highlights the discussant’s altruistic goal of bridging gender gaps in occupational settings and her decision to pursue a field where the impact could be more pronounced.

### Psycho-social wellbeing and stress

**Fig 2:**
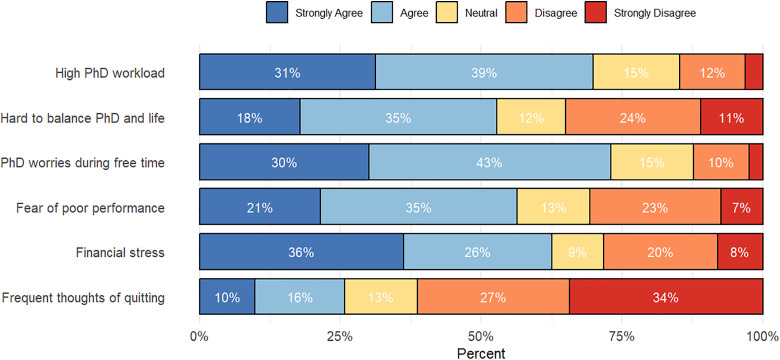
Perceived workload, financial stress, and emotional well-being among sampled women in STEM doctoral programs in sub-Saharan Africa (*n* = 163).

The online survey asked respondents to indicate their level of agreement or disagreement with statements related to feelings of stress and being overwhelmed during the PhD training. 70% of respondents reported experiencing an excessive workload, half found it difficult to achieve work-life balance, nearly three-fourths worried about their dissertation during their free time, and 62% experienced financial stress (Fig 2). Additionally, 26% reported frequent thoughts of terminating their PhD. Not shown in the figure are survey results on PhD completion: only 4% of the surveyed alumni did not complete their PhD, 11% temporarily paused their studies, and 85% successfully earned their doctoral degree.

Qualitative findings support the survey results related to financial stress, workload, and work-life balance. Women discussants did not, however, mention feelings of terminating the PhD. In two of the seven discussions, financial stress was highlighted. In one FGD, discussants mentioned that because of a lack of funding opportunities for postgraduate studies they must rely on their family members for financial support. In another FGD, participants explained that it is often not possible to get a paid leave of absence from work to pursue doctoral studies. This quote is illustrative of the latter point:

*“[My] employer said they don’t sponsor, they don’t pay PhD students, they don’t support PhD programs. So, if you take the program you are scrapped from the payroll. So, I declined, several of us declined the study leave without pay.”*

In this instance, the speaker has a master’s degree but refuses to take unpaid leave from her job to pursue a PhD, because she cannot or does not want to give up income. Indirectly, this speaker seems to be pointing to two realities: the lack of funding for PhD students from universities and a clear message from employers that additional training is not sufficiently valued to keep full-time PhD candidates on the payroll.

Time stress came up as important in the FGDs. Some women talked about the heavy time commitment required of postgraduate students in their field.

*“Sometimes there are many hours of lessons you can do from 8h to 13h, from 15h to 20h. There is no time to assimilate. It’s quite heavy. We’re tired, we don’t have time to do practical work once on the social campus and there are plans to do tutorials to prepare and in the end it’s a lot. We don’t have time to prepare everything.”*

The discussants also compared themselves to women postgraduate students in other fields and felt their own time pressure was far greater.

*“The women are in the other fields because there are less than 4 hours of lessons per day so there is less stress, they are relaxed… while we don’t even have time to braid our hair, and that’s the difference.”*

In most of the FGDs, women spoke candidly about the challenges they face balancing postgraduate studies with their other roles, such as that of wife or mother. The two quotes below drawn from discussions in each of the three countries articulate this point well:

*“When you have a family, you have children to take care of, you have a husband and many times usually you see when the husband is studying everybody is like don’t go to that room daddy is studying, don’t disturb even the kids know but now when it is a lady (others giggle), she is supposed to really cook or rather organize the people to cook and the children are all over her.”*

*“There are also other factors like marriage, early pregnancy and then household chores because in our society, it is women who do these things. It’s a little bit difficult to do research with all these roles that await us at home.”*

The speakers point to two issues that affect women in heterosexual relationships: the perception that women’s time is subject to interruption whereas men’s is not and the social expectations that women do more housework and childcare than men. Anecdotally, authors of this study have been party to discussions about the substantial presence of single women among the ranks of African female scientists and the benefits being single offers for career advancement alongside the downsides, such as social stigma and not having a family.

### Self-efficacy

Self-efficacy was assessed in the online survey with the use of two questions. First, respondents were asked to respond on a five-point Likert scale about whether they have what it takes to succeed as a PhD student, following a question asking them to attribute percentages of success in their discipline to factors such as intelligence or talent, hard work, educational preparation, networks, and luck. Second, respondents were asked whether they felt educationally prepared when they commenced PhD training.

Although not included in the figures, survey responses indicate that most participants expressed confidence in their ability to succeed as PhD students, with 54% strongly agreeing and 41% agreeing. Only a small percentage were less confident, with 2% disagreeing and 2% neither agreeing nor disagreeing. However, perceptions of educational preparedness were more mixed. Over half of the respondents (55%) strongly disagreed that they were educationally ready when they began their PhD program, with an additional 7% disagreeing. In contrast, 29% either agreed or strongly agreed that they had felt prepared.

The FGDs provide a nuanced perspective on self-efficacy, highlighting three key themes: self-efficacy evolves over time, confidence derives partly from a belief of possessing natural aptitude for science and math, and determination matters importantly for navigating challenges and achieving success in STEM PhD programs. Discussants explained that, while they initially experienced self-doubts, they eventually overcame these doubts, which fostered empowerment:

*“We are the ones who limit ourselves sometimes. In my experience when I was applying for the job for being an instructor in electronics dealing with lab and electricity, you would get sometimes electrocuted, I was one among men. So, we went for interview I was one among two, three then we talked and talked then I was like I cannot be considered among them considering the task that I have to handle, I cannot be chosen. Fortunately, I got chosen and they called me and I was kind of surprised because they had asked me how will you handle this with your hands but I said I will handle it and I still handle it.”*

In this instance, the speaker cites two factors that influence her experience. First, the electronics work she is invited to do involves some adjustment. Experimental laboratories by their very nature are places where the unexpected, such as the possibility of electrical shock, is hard to avoid. At the same time, while electrical shock is commonplace in this setting, this speaker, as one woman among many men, initially sees her gender as a disqualifying event. Specifically, she perceives that while men can endure occasional electric jolts, no woman could. Ultimately, she proves otherwise, managing just as her male colleagues do, and this may be a source of empowerment.

As far as confidence partly deriving from natural aptitude, several women shared how they recognized their strong abilities in math and science from an early age, as illustrated by this quote:

“*I was good in sciences right from primary I loved math, I loved sciences so it was easier to grasp concepts in sciences, than this science, what is it called the other subjects where you had to memorize*.”

The third self-efficacy theme emerging from the FGDs is strong determination and its role in overcoming barriers. One discussant exemplified this by expressing confidence in her ability to complete a graduate degree in an IT program, despite the high likelihood of failure:

“*When I started masters, I started hearing that finishing is almost zero, am not going to finish this thing. I said I want to change the story.”*

### Family support and societal pressure

**Fig 3:**
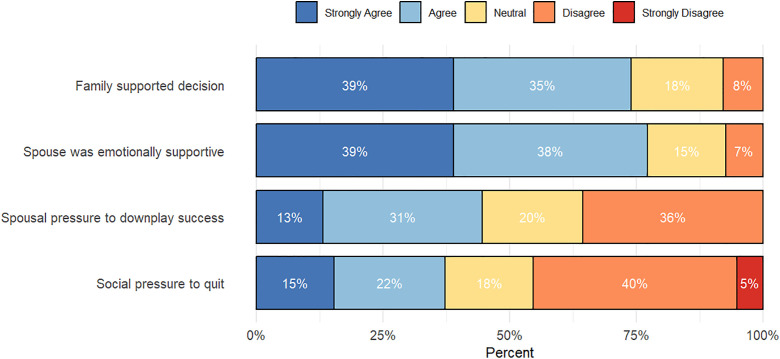
Perceived family support and societal pressure among sampled women in STEM doctoral programs in sub-Saharan Africa (*n* = 163).

The online survey measured family support and societal pressure by asking respondents their degree of agreement/disagreement to several statements. As shown in Fig 3, sampled women generally felt supported by their family and partners both for the initial decision to pursue a PhD and during the training period. However, responses also reveal that 44% of women felt pressure to downplay their achievements and career prospects to avoid issues with their spouse and 37% felt societal pressure to abandon their studies, as careers in science, engineering, and technology are often seen as incompatible with marriage and parenthood. It is important to note that depending on the question, 6% to 25% of respondents did not answer, as the questions referenced in Fig 3 were not applicable in some cases, such as when a respondent was unmarried.

FGD responses generally support the online survey results. Several participants described the moral and financial support offered by their family and the advantages such support offers:

*“I am not facing that many challenges [as others are facing] … because my husband understands why I should study and my family, my mum especially… I am fine, though it is challenging but I am okay.”*

*“Really, I have no pressure because my husband does not put pressure on me. He tells me to continue my studies until I finish.”*

*Moderator: “Who pays for your studies?” Discussant 1: “Our parents. It’s my father who pays.” Discussant 2: “For Me, it’s my brother.”*

On the other hand, the FGD participants conveyed an understanding of how hard it can be to pursue postgraduate studies without the support of family. For instance, in one FGD when discussants were asked if women receive emotional support from family, they said it depends and went on to explain what this means:

*Respondent 2: “Because some of us were not even allowed to come here” Moderator: really, why didn’t they want you to come here?” Respondent 3: I think it is background, they have family, kids”*

Other women described pressure from their parents, especially mothers, to put their studies on hold and start families first. For example, in one of the FGDs, a participant shared, *“I want to continue and do the thesis, but my mother does not want, so she says that it is time for you to get married and you are starting to get old.”*

Consistent with the online survey, societal pressure and expectations strongly emerged in several focus groups as illustrated by these quotes:

“*Sometimes I keep wondering why I took physics. At my age, am expected to take care of people at home and still go to work and women just like to work on anything.”*

*“It’s the community that says it. We are told: You are not going to find a husband because the boys are afraid of women who have done their doctorate. And you there if you wait until you get your doctorate, you won’t have a husband.”*

*“You have a lot to do in the society in Africa. After you reach 18 or 20 years old you have to stop your studies and you already start doing other things to work, to be a woman who works for the education of children and all that. These are all things that push girls to drop out of school.”*

### Role models and mentors

**Fig 4:**
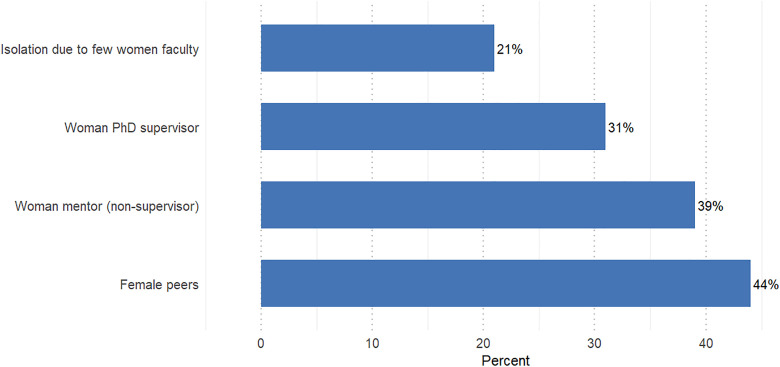
Perceived isolation, female role models, and gender representation among sampled women in STEM doctoral programs in sub-Saharan Africa (*n* = 163).

The online survey included questions about female role models, mentors, and peers. Among the PhD alumni surveyed, 21% reported feeling isolated because of a lack of female faculty, 31% had a female PhD supervisor, 39% had a female mentor (other than their PhD supervisor), and, on average, 44% of the respondents’ PhD peers were women (Fig 4).

While the online survey suggests male dominance in the STEM departments studied, the degree of this dominance appears greater in the FGDs, where, for example, discussants expressed how uncommon it is for them to have women supervisors.

*“We have male mentors because there are no females who are maybe doing that specific course around, so in a ratio of 1:10, you find one lady ten men so you have to rely on them but with time we are trying to take up this courses so that we can be many and we can actually mentor the other ladies.”*

Whereas the survey stopped short of exploring whether women students have a demand for women role models and mentors, the FGDs investigated the issue and revealed that women were very divided on this matter. On the one hand, many of the women expressed a strong interest to work with women advisors and mentioned certain advantages this offers, such as greater understanding and compassion on personal matters like pregnancy or marital issues. Two quotes illustrate this clearly:

*“I think increasing females in the sector of employment because it is easier even to discuss with a female supervisor, your things even personal stuff. She becomes like a mum to you.”*

*“Like the female ones, you can easily fit and identify with them and maybe when you reach a point that you need some personal assistance it’s easier to relate with them. But for the male ones, they have to push and they might not see behind the curtains of what you are going through to achieve your dreams but they can really push you and even assure you that you have the potential.”*

It is notable that the issue being discussed here, how would a female advisor respond to female student needs, may reflect wishful thinking rather than lived reality. In the near total absence of female professors, it would be easy to idealize what a female role model could be like, as expressed above. Whereas when women do begin to achieve leadership roles, it emerges that they vary in their relationships with junior women. An interesting follow-up question would be how these students imagine they will treat their students should they become professors or advisors themselves.

To some degree the data indicated that female solidarity was perhaps already much rarer than respondents were willing to admit. For example, it was not uncommon for the women discussants to express a preference for male lecturers and supervisors. For instance, in one FGD a discussant said she preferred having a women supervisor, while two of her peers expressed a preference to work with a male. In another FGD, three women stated a preference to have a male (vs. female) supervisor, the fourth woman in the group had no gender preference. The discussion of this issue in the latter FGD began like this:

Moderator: *“Do you prefer to have male lecturers over female lecturers?”* Discussant 1: *“I prefer male lecturers.”* Discussant 2: *“Male lecturers.”* Moderator: *“How about you?”* Discussant 3: *“Me too.”* Moderator: *“Why do you say so?”* Discussant 1: *“I find that women are too complicated.”* Discussant 2: *“Yes they are complicated.”*

The same discussion ended like this:

Moderator: *“I thought that female lecturers were more patient?”* Discussant 2: *“Here it’s the opposite. Fortunately, there are not many of them.”* Discussant 1: *“Yes, we don’t have many.”* Moderator: *“So you are more comfortable with male lecturers?”* Discussant 1: *“Yes, sure, they are very nice.”*

Furthermore, women in several FGDs believed women faculty are sympathetic on personal issues but competitive on academic matters. For instance, one discussant stated a sentiment shared by her focus group:

*“So, when women get to that point especially those who are older because they lived different times when science had very few ladies they have that mentality of they want to be the only ones here so they feel we are in competition but when you go to them with issues like family, marriage they are supportive because that is not competition.”*

### Departmental culture: stereotypes and discrimination

**Fig 5:**
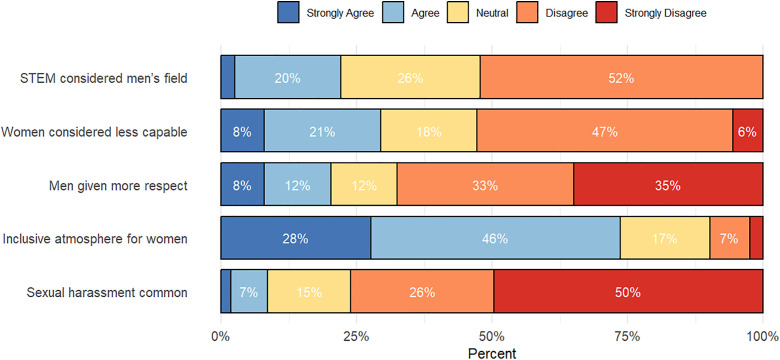
Perceived gender bias, exclusion, and sexual harassment reported by sampled women in STEM doctoral programs in sub-Saharan Africa (n = 163).

The online survey included several questions to assess the presence of gender-based stereotypes in STEM PhD programs. Fig 5 presents percentages for the degree of disagreement or agreement with these statements. The figure indicates that a prevalent stereotype was that women are less capable than men in science, with 29% of respondents agreeing or strongly agreeing. Additionally, slightly more than 20% perceived a stereotype that science is a men’s field, and 20% of respondents felt that men received greater respect in science. Furthermore, 26% of respondents reported a lack of gender inclusiveness in their PhD program.

In the five FGDs where sexism and gender stereotypes were discussed, participants were quite vocal, sharing experiences of how they are devalued and marginalized because of their gender. It is not uncommon, for instance, for women to be the brunt of jokes or criticized, as illustrated below.

*“I have experienced that at work because at my work in the IT department I was the only female so they were making jokes and saying something bad about females, and I am like this is not funny.”*

*“For example, it has always been said that women scientists are not like other women, by the way they dress, and they behave... for example computer scientists, mathematicians. They are not at the top, they do not have the class, they are so busy they do not have the time, and then they are all the time with the men... Well these are prejudices.”*

In support of the online survey findings, the stereotype that females are not as capable as males seems to exist in the postgraduate programs studied, as illustrated here:

*“And when I tell my students am teaching physics, they are like, it’s not possible’. They could not just believe that a female can teach physics.”*

*“In group discussion, the men always say, ‘no matter what you do definitely there is no way you will overtake us.’ I don’t know if they mean generally or socially academically.”*

“*Yes, sometimes we hear that this area is for boys… Because they think it’s difficult and that we don’t have the capacity to do it… They say it’s demanding especially for women who have to stay at home and take care of children and the house.”*

The latter quote suggests that some of our discussants’ male peers and faculty consider postgraduate studies as unsuited to women with children. This viewpoint is reinforced in the following quote from another discussant:

*“Like in physics department, the male staff there have phobia for pregnancy. For instance; when I enrolled for my postgraduate, they said, ‘thank God you are a nun’ and one day one of them told me that, ‘I hope sisters do not get pregnant’ and his explanation was that most ladies get scholarships but when they get pregnant, they “disappear.” This was to an extent that he called one of the lady’s husband and told them not to impregnate their student.”*

The concern about the time that children take away from academic endeavors is certainly not an exclusively African issue. At the same time, a professor contacting the husband of a student to discourage family formation would not be acceptable in many contexts.

### Sexual harassment

About 9% of surveyed women agreed or strongly agreed that sexual harassment by faculty was common in their PhD program (Fig 5). Not shown in the figure is that less than half (45%) were aware of a sexual harassment policy at their PhD institution, and only 54% were familiar with the university’s reporting mechanisms for sexual harassment cases.

In three of the FGDs, the discussants were quick to say that there is no problem of sexual harassment in their department and the conversation moved to another topic without probing by the moderator. In the other four FGDs, a general pattern was one in which the discussants mentioned some instances of sexual advances by male faculty, but they did not consider this necessarily to be sexual harassment although in many contexts these situations would have been considered as such. The quotes below, one from each of three FGDs are illustrative:

*“I have never experienced it nor ever heard of any case like that. Some lecturers may hit on you but they would not go to the extent of sexual harassment. In our context, some lecturers may even propose to marry you. A colleague of ours got married to one of our lecturers. Therefore, we do not look at what they do as sexual harassment.”*

*“I would not say it is harassment per se but I feel like sometimes people want to flirt with you just because you are female or having something you know maybe I am offering IT support and you are having that…, so they want to flirt with you and the guy thinks you will do something.”*

*“It is happening, actually when I was at the undergraduate, I saw a lecturer enters somebody’s, the student’s room. But maybe it’s within, the student also accepted, its collaboration if it happens.”*

In one FGD, sexual harassment was talked about at great length, with discussants indicating it is a problem in their department but mainly for younger, unmarried women and MSc students. One discussant shared a personal experience of being harassed,

*“I am young so you go there he starts your discussion in terms of academics then in the middle of the conversation things change so you have to like balance because you cannot offend this person. So, you have to stick somewhere and you don’t show anything negative according to what he is trying to get in the conversation but you also focus on what took you there… I had to change one of the supervisors. I forced the chair to change him… Personally, I don’t feel like changing my career but I felt like what is happening, you know this person is like your dad, you see why can’t he think beyond, it is like you don’t even want a male supervisor anymore.”*

FGDs suggested that reporting cases of sexual harassment is fraught with difficulties. On being asked if reporting mechanisms work, participants said sexual harassment was rarely reported and offered these explanations:

*“They work but it is all about reporting. How do you report something to a male department chair that what another male is, maybe he is doing the same thing? So, it doesn’t even make sense.”*

*“I think one of the factors that makes students not report sexual harassment is that if some of these cases involve lecturers with high rank within the university then it makes it difficult for one to file a report and the story only spreads among the students. And within the university, there are laws set to deal with such issues but most of the students are not aware of such laws.*”

The discussant who shared her personal experience of sexual harassment did not feel she could report the incident, even to a female faculty member.

*“I just told her I am working with somebody else so I want my supervisor changed. So, mostly you just send your work through email, even if you have something that you could discuss face-to-face it is now difficult, you cannot go because there is something.”*

## Discussion

### Synthesis of the study findings

This study challenges the dominance of Global North narratives in STEM higher education by focusing on the experiences of women in STEM at African institutions and highlighting African universities as sites of innovation and resilience.

The study explored women’s challenges and enablers in STEM postgraduate education through an online structured survey of PhD alumni of 40 African universities in 17 countries (*n* = 163 women) and FGDs with 39 postgraduate students attending universities in three African countries.

### Study findings on gender-based challenges in STEM

We found that while African women face challenges similar to those encountered by their counterparts in other regions, these play out in contextually specific ways. Over 60% of female PhD alumni surveyed reported financial stress during their doctoral studies. In FGDs, participants described how inadequate university funding forces students without family support to abandon their studies, often at the MSc level. These findings align with global research on women in STEM [23,24], while studies in Africa document how economic and sociocultural factors compound these barriers. For instance, research in SSA suggests that limited funding is further exacerbated by perceived biases in allocation, lack of transparency in selection processes, and delays in disbursement [25].

Beyond financial challenges, unequal time commitments significantly hinder women’s academic progress, consistent with feminist literature on housework, the second shift, and women’s time constraints [26]. Most of the sampled women reported excessive PhD workloads and difficulty balancing studies with personal life. FGDs revealed that caregiving and household responsibilities often prevent women from earning additional income, which could ease the financial challenges of PhD training, or dedicating consistent blocks of time to their research, leading to delays in degree completion. These barriers to progression have been documented in other African studies of women studying [18] or working in STEM [17,27].

Female supervisors and mentors can play a critical role in supporting women’s progression in STEM fields by serving as role models, countering negative gender stereotypes, and fostering more inclusive academic environments [9,28–30]. The lack of visible role models for young female researchers is a persistent concern. [31] documents the importance of exposure to relatable role models: women a few career steps ahead who have successfully balanced academic work with family life.

However, our findings challenge the assumption that gender concordance ensures supportive mentoring. While some FGD participants expressed appreciation for female supervisors who understood gendered challenges, most reported a preference for male supervisors. Several described female supervisors as overly competitive or “complicated,” suggesting unmet expectations of solidarity or perhaps disappointment when senior women were unable or unwilling to support them.

This pattern may reflect broader institutional and cultural pressures: women navigating male-dominated academic environments may adopt protective strategies that limit their accessibility to junior women, due to time constraints, role overload, or concerns about perceived favoritism [32,33]. In such contexts, mentoring is shaped more by institutional hierarchies and norms than by gender identity. These findings clarify the need for structured, intentional mentoring programs that emphasize communication, integrity, and responsiveness to students’ diverse life circumstances and definitions of success [34].

Societal expectations emerged as another main barrier [32]. Surveyed women and FGD participants described cultural attitudes that pressure women to prioritize family responsibilities over academic ambitions. These pressures often frame advanced degrees and STEM careers as incompatible with traditional roles as wives and mothers. For many women, completing a PhD in this context represents not only an academic milestone but also an act of resistance against restrictive gender norms.

Sexual harassment was reported as less pervasive in this study than suggested by other research [34–36]. However, FGDs revealed that cultural norms often normalize inappropriate behaviours, leading women to underreport incidents or view them as tolerable. This normalization, shaped by cultural norms and power dynamics, can lead women to downplay or internalize inappropriate behavior as a tolerable part of academic life, rather than a violation of their rights.

### Study findings on women’s resilience in STEM

Findings suggest that resilience stems from a combination of passion for science, self-efficacy, an unwavering determination to persist, a sense of realism about institutional barriers, and strong family support. The online survey results suggest that African women students, like their counterparts in Europe and North America [37,38], are deeply passionate about STEM and view science careers as pathways to social status and job security. Survey responses indicated moderate levels of self-efficacy, defined as belief in one’s capability to succeed in a specific domain [39]. FGDs added nuance, revealing that self-efficacy is dynamic rather than fixed. Participants spoke of an early recognition of their aptitude in math and science, but they also described periods of self-doubt that were overcome through their determination not to be blocked by sexist attitudes about women in science. Discussants expressed an awareness of the socially constructed hurdles they face in their chosen career pathway, including unwelcoming academic environments, excessively demanding curricula, and deeply rooted gender stereotypes. Yet they also demonstrated confidence in their ability to succeed, articulating a desire to “change the story,” as one FGD participant put it. As [40] observe in interviews with health researchers in five West African countries, women in STEM often perceive themselves as equally competent as their male peers and reject the gender discrimination they face.

A sense of realism emerged from the FGDs and appears to be a factor that prevents the women participants from getting overly frustrated and dropping out of their PhD studies. Discussants described themselves as in the minority among male peers and faculty but only 21% of online survey respondents reported feeling isolated. Discussants spoke candidly about encountering gender stereotypes and their objection to sexist jokes and prejudicial remarks. In the survey, women mentioned thoughts of quitting their PhD, but in the FGDs, no one expressed serious consideration of this. Similarly, it was surprising how casually women discussed the matter of sexual harassment. That some were able to laugh about this very sensitive topic speaks to a sense of resilience and acceptance.

Family support was also identified as critical to persistence in STEM [13]. Discussants highlighted how financial and moral support from parents, spouses, or siblings alleviated key challenges presented by PhD training and helped them withstand societal pressures to marry early, have children, or choose careers that are less demanding than STEM. Women without such support often questioned the wisdom of their decision to pursue postgraduate studies.

In summary, African women in STEM postgraduate programs face substantial and multifaceted challenges, including financial constraints, lack of role models, unequal time burdens, cultural expectations, and sexual harassment. These women exemplify resilience, achieving remarkable success despite systemic barriers. Their experiences provide lessons on navigating male-dominated fields, while highlighting the continued need for institutional and sociocultural reforms.

### Policy implications

Drawing on our study findings, several priority areas emerge for African universities, governments, and international funders seeking to promote gender equity in STEM postgraduate education. These can be grouped into three overarching objectives: improving the postgraduate experience of women in STEM, supporting women in achieving their personalized career goals, and facilitating timely PhD completion and transitions to the workforce.

### Improving the postgraduate experience of women in STEM

Family-friendly policies are essential to supporting women’s retention and well-being in STEM postgraduate programs. For example, the Consortium for Advanced Research Training in Africa (CARTA) covers the full costs of women doctoral fellows who are breastfeeding mothers to bring their child and a babysitter along for a month-long residential training seminar [41]. The program also allows fellows to stop the funding clock during their maternity leave, if they request it, with funding resuming upon their return to doctoral studies. University-based childcare centers, such as those supported by Senegal’s “Case des Tout-Petits” initiative, also demonstrate scalable models for reducing strain on women balancing study and caregiving responsibilities. These supports should be made visible to students through university orientation programs and gender units. Male champions can further contribute by normalizing shared caregiving and household responsibilities, reinforcing the message that marriage and motherhood are compatible with scientific careers.

### Supporting women in achieving their personalized career goals

Tailored mentorship is critical to women’s persistence and fulfillment in STEM. Programs like African Women in Agricultural Research and Development (AWARD) and the HIGHER Women Consortium offer tested approaches [42,43]. As [42] note, matching a fellow with her best mentor is an art, requiring both commitment and clear selection criteria, such as a mentor’s recognition in the field, the fit between the mentor’s and mentee’s research interests, proximity, and the mentor’s demonstrated empathy and leadership. Evaluations show such mentoring programs improve mentees’ confidence and research performance while promoting gender responsiveness among mentors, particularly men, increasing their potential as institutional change agents [42,43].

Funders and institutions should also support women’s participation in regional and global scientific forums that feature female keynote speakers and celebrate women’s contributions. Role models must reflect the diversity of women’s life circumstances, including single women, childfree women, and others who do not conform to dominant gender norms about marriage and family.

### Facilitating timely PhD completion and career transitions

Financial support is indispensable for women navigating the rigors of STEM postgraduate education. Programs like RSIF, which reserves a minimum of 30% of awards for women (with a 50% target), and Kenya’s Mawazo Institute, which combines funding with skills training and public engagement, help reduce financial burdens and increase visibility.

Greater flexibility in program design is also needed. Sandwich programs should accommodate scholars with young children through shorter durations or remote participation. Institutions should adopt policies that allow for study interruption and reentry in response to major life events. Such flexibility is essential to retaining talented women, especially in disciplines with few female faculty and limited role models.

Taken together, these policy interventions can support more inclusive, equitable, and effective systems of STEM postgraduate training across Africa.

## Study limitations

This study has several limitations. First, the survey used non-probability sampling and included a relatively small sample of PhD alumni, many from World Bank–funded African Centres of Excellence (ACE) programs. These programs often offer stronger infrastructure and financial support than typical doctoral programs, and participating alumni may be disproportionately high achieving. This limits the generalizability of our findings to the broader population of African STEM postgraduate programs, particularly those with fewer institutional resources.

Second, the survey and focus group participants were drawn from different populations. Surveyed alumni, whose responses reflect retrospective experiences across diverse African contexts, were not the same individuals as the current MSc and PhD students who participated in FGDs. This limits direct comparability between data sources.

Finally, qualitative data collection was affected by the sudden passing of lead qualitative researcher Dr. Sheila Onzere at the start of the data collection process, a loss to both this project and the broader research community. An experienced research consultant and her team subsequently led the FGDs. While the transition was managed effectively, some variation in facilitation styles affected consistency across transcripts.

## Implications for research

This study reveals several areas that merit further investigation. First, while structured mentoring programs like AWARD and the HIGHER Women Consortium have demonstrated positive short-term outcomes, the long-term impacts of these programs remain under-researched. There is a need for longitudinal studies that track women’s career trajectories following participation in structured mentoring, including effects on STEM career retention and leadership attainment. Such research could also explore how women’s experiences with mentoring evolve over time and how different models—peer mentoring, intergenerational mentoring, or mixed-gender mentoring—may influence academic persistence and professional advancement.

Second, there is a pressing need for rigorous evaluations of family-friendly policies and gender-responsive institutional reforms in diverse African higher education settings. Research should investigate how interventions such as parental leave accommodations, on-campus childcare, flexible program structures, and gender equity training for faculty and administrators affect women’s postgraduate trajectories. Randomized controlled trials (RCTs) can provide evidence of the causal effects of such interventions, while comparative case studies can help identify the contextual and institutional factors that shape implementation and outcomes. Together, these approaches can inform the design of more effective and scalable models for promoting women’s persistence, well-being, and success in STEM postgraduate programs.

Further research could also explore how female scientists at different life stages in Africa navigate the trade-offs between career advancement and personal responsibilities, particularly in male-dominated STEM environments. Such studies would offer valuable insights for policies that support inclusive career pathways and work-life balance strategies beyond the PhD phase.

Finally, future research should take a more intersectional approach by considering how factors such as disability, family status, socioeconomic background, and geographic location shape women’s experiences in STEM postgraduate programs. This would support the design of more inclusive policies and interventions that respond to the diverse realities of African women in science.

### Notes

1. It is important to mention that we use the essentialist designations of man and woman in this study, because it reflects the accepted categories in the countries under study. In all the included African countries, gender non-conforming or non-normative identities are socially unacceptable and to varying degrees illegal. Binary gender norms are both the product of colonial legal structures and the ongoing resistance of Christian and Islamic religious communities.

## Supporting information

S1 Appendix RSIF gender study online survey questionnaire. (DOCX)

S2 Appendix RSIF gender study focus group discussion checklist. (DOCX)
