# Supplementary material for: Changing the narrative: Resilience of women in STEM in sub-Saharan Africa and institutional innovations to advance equity
Source: PLoS One. 2026 Jan 21;21(1):e0338973. doi: 10.1371/journal.pone.0338973 (PMC12822989; doi:10.1371/journal.pone.0338973)
Supplement: S1 Appendix — (DOCX) [file pone.0338973.s001.docx]

**S1 Appendix.** **RSIF gender study online survey questionnaire**

**Enhancing Participation in PhD Programs and Research in Science, Technology, Engineering, and Math (STEM) fields at African Universities**

**Former Student Questionnaire, March 2020**

**INFORMED CONSENT**

This research project is being conducted by the International Centre of Insect Physiology and Ecology (*icipe)* under the Regional Scholarship and Innovation Fund (RSIF). The study seeks to learn about the experiences of PhD students in science, technology, engineering, and math (STEM) fields in Africa. Completion of this survey will take about 15-30 minutes. Your participation is completely **voluntary**. You have the right not to participate or withdraw from the study at any time. Information you provide is for research purposes only and will be confidential. There are no foreseeable risks or discomfort from your participation. Although you will not directly benefit from taking part in this study, your participation will help improve knowledge of the barriers and supports to PhD students in scientific fields, so that we can recommend interventions to increase the numbers and experiences of women and men in STEM in Africa.

Do you agree to participate in this study? Your continuation of the survey and checking “Yes” will be taken as willingness to participate.

1. Yes
2. No

#### Please contact [Project Lead] if you have any questions or concerns.

#### Part A. Details of the PhD training and demographic and socio-economic status

#### Please remember back to the time when you were a PhD student and answer some questions about that point in your life.

#### A1. At what university did you pursue a PhD? _________________________

#### A2. In what PhD program/department did you pursue the PhD?

#### Climate change

#### Food security

#### Physics

#### Life sciences

#### Computer science

#### Engineering

#### Other (specify) _________________

#### A3. In what year did your enrollment in this PhD program begin? _______________

#### A4. In what year did your enrollment in this PhD program end? _______________

#### A5. Which of the following situations best corresponds to yours?

#### I completed the PhD program

#### I have temporarily interrupted my PhD studies

#### I did not complete the PhD program

#### A6. Did your program or university have a time limit for PhD completion?

#### Yes [*continue to A7*]

#### No [*skip to A9*]

#### A7. In your program/university, how many years was allowed for completing the PhD? ______ years

#### A8. Was the time limit for PhD completion enforced?

#### Yes

#### No

#### A9. When you began the PhD program, did you feel that you had the necessary educational preparation to succeed as a PhD student?

#### Strongly disagree

#### Disagree

#### Undecided

#### Agree

#### Strongly agree

#### A10. During your PhD training, did you submit any proposals for competitive research grants?

#### Yes [*Continue to question A11*]

#### No [*Skip to question A12*]

#### A11. Were any of these proposals funded?

#### Yes

#### No

####

#### A12. During your PhD training, how many papers did you have accepted for publication in peer-reviewed journals? ___________

#### A13. During your PhD training, were you involved in teaching a course (i.e., as instructor or co-instructor)?

#### Yes

#### No

#### A14. During your PhD training, how many times did you present a poster or paper at a national or international conference? ____________

A15. Did you have a partner/spouse at the time of your PhD training?

#### Yes [*Continue to A16*]

#### No [*Skip to A17*]

#### A16. What was your partner/spouse doing at the time of your PhD training? Please check all that apply.]

1. Unemployed
2. Working in a STEM field
3. Working in a non-STEM field
4. Pursuing an undergraduate degree
5. Pursuing a graduate degree
6. Other (specify) _______________

#### A17. During your PhD training did you have any child(ren) under the age of 5 years?

#### Yes [*Continue to A18*]

#### No [*Skip to A19*]

#### A18. What sort of childcare arrangement did you have during your PhD training? [*Check all that apply.*]

#### Spouse/partner took care of child(ren)

#### Other family member took care of child(ren)

#### Free or subsidized childcare

#### Paid for childcare out of pocket

#### Other (specify) _____________________________

#### A19. Which, if any, of the following life events did you experience during your PhD training? [Check all that apply.]

#### Entered into a marriage or marriage-like relationship

#### Divorce/separation or dissolution of a marriage-like relationship

#### Birth/adoption of child(ren)

#### Death of immediate family member or friend

#### Assumed primary responsibility for a person with illness or other disabling condition

#### Major illness (mental or physical) or other disabling condition

#### Loss of job

#### None of the above

A20. What was the most important source of funding for your PhD training?

#### Parents

#### Partner/spouse

#### Other family members

#### Self (own savings or income)

#### University funding

#### Non-university funding

#### Other (specify) ___________________

#### A21. What was the second most important source of funding for your PhD training?

#### Parents

#### Partner/spouse

#### Other family members

#### Self (own savings or income)

#### University funding

#### Non-university funding

#### Other (specify) ___________________

#### A22. During your PhD training, did you hold a job that was unrelated to your PhD research?

#### Yes

#### No

#### A23. Thinking back to your childhood, would you say you had an early interest in STEM?

#### Strongly disagree

#### Disagree

#### Undecided

#### Agree

#### Strongly agree

#### Part B. Motivation and socio-psychological well-being during your PhD training

The following is a list of some possible factors that motivate students to persist in their doctoral studies. Please indicate by checking the appropriate box the extent to which each statement corresponded to your own situation during your PhD training.

| Motivation type | Strongly disagree | Disagree | Neither disagree nor agree | Agree | Strongly agree |
| --- | --- | --- | --- | --- | --- |
| B1. I felt passionate about my doctoral research project. |  |  |  |  |  |
| B2. I felt my doctoral training was improving my knowledge and skills in my field of study. |  |  |  |  |  |
| B3. It was important to me to contribute to the state of knowledge in my field of study. |  |  |  |  |  |
| B4. I had an excellent research opportunity (e.g., working on cutting-edge research or with a prestigious faculty member). |  |  |  |  |  |
| B5. I was motivated to persist with the PhD to avoid disappointing my advisor/supervisor. |  |  |  |  |  |
| B6. I was motivated due to commitments that I needed to fulfill (e.g., with funding agencies, employers, collaborators). |  |  |  |  |  |
| B7. I did not want to be perceived as a quitter. |  |  |  |  |  |
| B8. I had a strong personal goal for a timely PhD completion. |  |  |  |  |  |
| B9. I felt my doctoral studies would help me achieve my life goals and objectives. |  |  |  |  |  |
| B10. I was motivated by the prestige associated with a PhD. |  |  |  |  |  |
| B11. I was motivated by the future job prospects associated with holding a PhD. |  |  |  |  |  |

Below are some statements related to feelings of stress and being overwhelmed, which are not uncommon among graduate students. Please indicate by checking the appropriate box the degree to which you disagree or agree with these statements.

| Socio-psychological well-being indicator | Strongly disagree | Disagree | Neither disagree nor agree | Agree | Strongly agree |
| --- | --- | --- | --- | --- | --- |
| B12. My workload during the PhD training was too high. |  |  |  |  |  |
| B13. I found it difficult to balance my PhD studies with my personal life. |  |  |  |  |  |
| B14. I worried about my dissertation during my free time. |  |  |  |  |  |
| B15. I often feared that I was not doing well enough in my doctoral studies. |  |  |  |  |  |
| B16. I experienced financial stress/difficulties during my doctoral studies. |  |  |  |  |  |
| B17. I frequently felt like terminating my PhD studies. |  |  |  |  |  |

#### Part C. Support networks, societal factors, and perceptions

#### These questions ask about your perceptions of what it takes to be a successful doctoral student in your discipline and how different people in your life and societal messages influenced your decision to pursue a PhD or your experience as a graduate student.

#### C1. To succeed as a PhD student in your discipline, what do you think matters most? Please indicate the percentage of success that you attribute to the following factors: [*The percentages for items 1 through 5 should sum to 100%*]

#### Intelligence or talent ________ %

#### Hard work ________ %

#### Educational preparation ____________ %

#### Networks (i.e., who you know or work with) ___________ %

#### Luck (e.g., landing opportunities simply by being in the right place at the right time) ____________ %

#### Now, please indicate by checking the appropriate box the degree to which you disagree or agree with the following statements.

|  | Strongly disagree | Disagree | Neither disagree nor agree | Agree | Strongly agree | Not applicable |
| --- | --- | --- | --- | --- | --- | --- |
| C2. You have what it takes to succeed as a PhD student. |  |  |  |  |  |  |
| C3. Your parents/guardians and siblings supported your decision to pursue a PhD. |  |  |  |  |  |  |
| C4. Your partner/spouse was emotionally supportive during your PhD studies. |  |  |  |  |  |  |
| C5. During your PhD studies, you felt pressure to downplay your achievements and career prospects to avoid issues with your spouse (e.g., making her/him feel insecure)? |  |  |  |  |  |  |
| C6. During your PhD training, you felt societal pressure to abandon your studies because being a scientist/engineer/techie is considered incompatible with being married and having children. |  |  |  |  |  |  |

#### Part D. Role models and mentors

#### This section asks about your personal experiences with role models and mentors during the PhD training. Please check the appropriate box.

|  | Strongly disagree | Disagree | Neither disagree nor agree | Agree | Strongly agree |
| --- | --- | --- | --- | --- | --- |
| D1. Your PhD program had fewer female professors than male professors. |  |  |  |  |  |
| D2. You felt isolated because of the lack of women faculty in the PhD program. |  |  |  |  |  |
| D3. Your PhD advisor provided effective guidance in professional activities (e.g., writing articles for publication, conference presentation, networking)? |  |  |  |  |  |
| D4. Your PhD advisor was supportive of your personal career goals. |  |  |  |  |  |
| D5. Your PhD advisor was aware and appreciative of the value you brought to research projects. |  |  |  |  |  |
| D6. You met frequently or often enough with your PhD advisor. |  |  |  |  |  |
| D7. Your PhD advisor had an overall positive impact on your PhD training experience. |  |  |  |  |  |

#### D8. What is the gender of your main PhD advisor?

#### Female

#### Male

#### D9. During your PhD studies was there someone besides your PhD advisor that you considered as a mentor, either formally or informally, who positively impacted on your PhD training?

#### Yes (*continue to D10*)

#### No (*skip to next section*)

#### D10. What is the gender of this mentor?

#### Female

#### Male

#### Part E. Departmental culture: gender stereotypes and discrimination

#### This section asks about the culture in your PhD department/program. Please check the appropriate box to indicate the extent to which you agree or disagree with the statements below.

|  | Strongly disagree | Disagree | Neither disagree nor agree | Agree | Strongly agree |
| --- | --- | --- | --- | --- | --- |
| E1. In your PhD program, there was a stereotype that STEM fields are “men’s fields”. |  |  |  |  |  |
| E2. In your PhD program, women were considered less capable than men in scientific/technical endeavors. |  |  |  |  |  |
| E3. In your PhD program, women were considered less dedicated to work than men. |  |  |  |  |  |
| E4. In your PhD program, male students were given greater respect than female students? |  |  |  |  |  |
| E5. The classroom culture generally valued, included, and respected women students. |  |  |  |  |  |
| E6. In your PhD program, there was an atmosphere of women’s inclusiveness among faculty and students. |  |  |  |  |  |
| E7. You received help and/or support from other doctoral students during your PhD training. |  |  |  |  |  |
| E8. Sexual harassment by peers was a common occurrence for women students in your PhD program. |  |  |  |  |  |
| E9. Sexual harassment by faculty was a common occurrence for women students in your PhD program. |  |  |  |  |  |

E10. The year you entered the PhD program, how many female students were in your PhD cohort (include yourself, if you are female)? ___________________

E11. The year you entered the PhD program, how many male students were in your PhD cohort (include yourself, if you are male)? ___________________

**Part F. University/program policies and practices**

#### F1. Did the university where you did your PhD training have any policies and practices in place to support women graduate students? (Examples are maternity leave, on-site or subsidized childcare, extension of academic deadlines.)

#### Yes

#### No

#### I don’t know

#### F2. Did the university where you did your PhD have a sexual harassment policy?

#### Yes (*continue to F3*)

#### No (*skip to F4*)

#### I don’t know (*skip to F4*)

#### F3. Were you aware of the university’s reporting mechanisms for sexual harassment during your PhD studies?

#### Yes

#### No

#### F4. Was there an office at your university for students that specifically focused on gender, diversity, and/or social inclusion?

#### Yes (*continue to F5*)

#### No (*skip to next section*)

#### Don’t know (*skip to next section*)

#### F5. How often did you visit this office?

#### Never

#### Seldom

#### Sometimes

#### Frequently

#### Part G. Learning resources and infrastructure

#### Now we have some questions about learning resources. Please check the appropriate box below to indicate your disagreement or agreement with the statements below.

|  | Strongly disagree | Disagree | Neither disagree nor agree | Agree | Strongly agree |
| --- | --- | --- | --- | --- | --- |
| G1. The laboratories in your PhD program or department were well-equipped for student practicals. |  |  |  |  |  |
| G2. The library at your university was well equipped with up-to-date and relevant study and research materials. |  |  |  |  |  |

Did your PhD program or institution offer any of the following for doctoral students? If so, did you participate in or use any of the following?

| Offering | Not offered, or don’t know | Yes, offered, but I did not use/participate | Yes, offered and I used/participated |
| --- | --- | --- | --- |
| G3. Orientation program |  |  |  |
| G4. Training courses on oral communication and presentation skills |  |  |  |
| G5. Training courses on writing and scientific publishing |  |  |  |
| G6. Training courses on research methods |  |  |  |
| G7. Leadership training |  |  |  |
| G8. Training courses on teaching skills/methods |  |  |  |
| G9. Regular seminar series |  |  |  |
| G10. Job placement services |  |  |  |
| G11. Student associations |  |  |  |
| G12. Social events for students and faculty |  |  |  |

#### Part H. Background information on the respondent

#### In this last module, we have some questions about your demographic background and current circumstances.

#### H1. What is your age? _____________

#### H2. What is your gender?

#### Female

#### Male

#### H3. What is your nationality? ____________________

H4. What is the highest academic qualification you attained?

1. MSc degree
2. PhD degree
3. Other (specify) _______________

H5. In what year did you obtain your MSc diploma? _____________

H6. At what university did you obtain your MSc? _________________________

H7. What are you currently doing? [*Check all that apply.*]

1. Unemployed
2. Working in a science, technology, engineering, or math (STEM) field
3. Working in a non-STEM field
4. Pursuing a graduate degree in STEM
5. Pursuing a graduate degree in a non-STEM field
6. Other (specify) _______________

#### H8. Among your parents or guardians, what is the highest level of education achieved?

1. Less than high school degree
2. High school degree
3. BA or BSc degree
4. MA or MSc degree
5. PhD degree
6. Other (specify) _______________

#### H9. Lastly, I would like to know if you are willing to share your contact email, in case we would like to follow-up with you?

#### Yes (*continue to H10*)

#### No (*interview is over*)

#### Other (Please specify)

#### H10. Contact email: _______________________________

#### Thank you for your participation in our survey!
