# Supplementary material for: Changing the narrative: Resilience of women in STEM in sub-Saharan Africa and institutional innovations to advance equity
Source: PLoS One. 2026 Jan 21;21(1):e0338973. doi: 10.1371/journal.pone.0338973 (PMC12822989; doi:10.1371/journal.pone.0338973)
Supplement: S2 Appendix — (DOCX) [file pone.0338973.s002.docx]

**S2 Appendix. RSIF gender study focus group discussion checklist**

**Enhancing Women’s Participation in PhD Programs and Research in Science, Technology, Engineering, and Math (STEM) fields at African Universities**

**Postgraduate Student Focus Group Discussions, November 2019**

**ORAL INFORMED CONSENT**

Hello. My name is ___________________. I am working with the International Centre of Insect Physiology and Ecology (*icipe)* on a study about women’s participation in postgraduate programs and research in STEM fields in West and East Africa. The Regional Scholarship and Innovation Fund (RSIF) is one of the flagship programs of the Partnership for skills in Applied Sciences, Engineering and Technology (PASET), an initiative by African governments to address systemic gaps in skills and knowledge necessary for long-term, sustained economic growth in sub-Saharan Africa (SSA). The objective of the study is to generate the evidence base needed for recommending strategies to enhance women’s enrollment, performance, and completion of postgraduate programs in STEM fields in sub-Saharan Africa (SSA), with special emphasis on RSIF host universities.

I am very interested to talk with you about your experiences as a postgraduate student at __________. I estimate the interview will take about one to two hours. Your participation is completely voluntary. You have the right not to participate and you can choose not to answer any questions you are asked, and there will be no consequences. Our conversation and the information I obtain from you is for research purposes only, and I assure your confidentiality. There are no foreseeable risks or discomfort from your participation. Although you will not directly benefit from taking part in this study, the information you provide will help improve knowledge of the barriers women face as postgraduates in STEM fields, so that we can recommend interventions to respond to those limitations, ultimately increasing the numbers and experiences of women in STEM in SSA.

Do you have any questions for us? **<*Researcher: pause and respond to any questions raised, then continue with the following statement*>.**

Do you agree to participate in this study? *[If YES, indicate below that the oral informed consent has been obtained. Then proceed with the question below regarding audio recording. If the respondent refuses, thank them for their time and end the conversation*.]

- Oral informed consent received

Do you agree to be audio recorded? *[If YES, indicate below. If any of the participants responds “NO”, proceed with the focus group without recording.]*

- Consent to audio record interview received

Signature of researcher: Date: / /

Please contact [Project Lead] if you have any questions or concerns.

**RSIF GENDER STRATEGY FGD RECORD**

**FGD LOCATION / IDENTIFICATION:**

**# OF PARTICIPANTS: GENDER: F ______ M _______AGE RANGE:**

**INTERVIEWER’S NAME(S):**

**DATE:**

**LOCATION:**

- Ensure safe, quiet, neutral location for the FGD.
- Welcome, thank them for their time.
- Introduce yourself, ask participants to briefly introduce themselves.
- Carefully go through the informed consent in full, ensure understanding, allow time for questions or concerns to be shared.
- Obtain and document verbal consent for all participants prior to proceeding. Those who do not wish to participate are free to leave. Thank them for their time.
- “House rules”
  - Respect, allow everyone to speak, one person to speak at a time, everyone gives space for others to talk, all to share their opinion
  - Honest answers are needed
- Avoid own opinions or guiding answers
- Ensure, no one else listens to the FGD
- Thank everyone at the end of the FGD

| **Questions** | **Notes** |
| --- | --- |
| 1. How would you describe the current situation of women’s participation in STEM fields?   *Points of discussion:*   - *Number of women as compared to men (make distinction between faculty, administrators, MSc and PhD students, undergraduate students)* |  |
| 1. What would you say are the main reasons women/men choose to pursue graduate studies in a STEM field? (distinguish between MSc and PhD)   Points of discussion:   - *Interest in STEM* - *Career choice/advancement* - *Opportunity (e.g., scholarship)* - *Social support or pressure* - *Other* |  |
| 1. What factors either challenge or facilitate women’s/men’s enrollment into a MSc or PhD program in STEM?   *Points of Discussion:*   - *Institutional policies and practices (affirmative action, discrimination, implicit bias)* - *Socio-cultural [social influences; emotional support; family circumstances; social impact; prestige; role models/mentors]* - *Economic [funding]* |  |
| 1. How would you describe your experience of having women in the department [as peers and faculty members/staff]?   *Points of Discussion:*   - *Role they play in enhancing positive/negative student experience* - *Perception of their capacity as compared to men* - *Role models /mentors [numbers; preference between male or female]* |  |
| 1. What challenges have you and others you know faced in your field of study/department?   *Points of Discussion:*   - *Supervision* - *Instructional materials* - *Lack of role models/mentors* - *Gender discrimination* - *Equipment* - *Funding* - *Emotional Support [family, spouse, children]*   *NB/Please note if the challenge is unique to a specific gender* |  |
| 1. What are the gender stereotypes in your field of study?   *Points of Discussion:*   - *Perceptions of differences between women and men in ability, work ethic, performance* |  |
| 1. Do you or others you know face gender discrimination in your department?   *Points of discussion:*   - *Inappropriate language* - *Sexual harassment* - *Sexist jokes/comments* - *Bullying [peers and/or faculty members]* - *Other forms* |  |
| 1. How do the challenges affect your pursuit of further studies or career prospects in STEM fields?   *Points of Discussion:*   - *Interest/motivation* - *Self-concept* - *Advancement in further studies/career* |  |
| 1. Are there strategies /interventions that can increase women participation in STEM fields? Which ones offer most promise? Why?   *Points of Discussion:*   - *Funding* - *Policies* - *Networking* - *Role Models/Mentors* |  |
| 1. Is there anything else that we haven’t covered in this interview that you think is important? |  |
